# Lactic Acid Bacteria Protects *Caenorhabditis elegans* from Toxicity of Graphene Oxide by Maintaining Normal Intestinal Permeability under different Genetic Backgrounds

**DOI:** 10.1038/srep17233

**Published:** 2015-11-27

**Authors:** Yunli Zhao, Xiaoming Yu, Ruhan Jia, Ruilong Yang, Qi Rui, Dayong Wang

**Affiliations:** 1 Key Laboratory of Environmental Medicine Engineering in Ministry of Education, Medical School, Southeast University, Nanjing 210009, China; 2 Department of Preventive Medicine, Bengbu Medical College, Bengbu 233020, China; 3 College of Life Sciences, Nanjing Agricultural University, Nanjing 210095, China

## Abstract

Lactic acid bacteria (LAB) is safe and useful for food and feed fermentation. We employed *Caenorhabditis elegans* to investigate the possible beneficial effect of LAB (*Lactobacillus bulgaricus*) pretreatment against toxicity of graphene oxide (GO) and the underlying mechanisms. LAB prevented GO toxicity on the functions of both primary and secondary targeted organs in wild-type nematodes. LAB blocked translocation of GO into secondary targeted organs through intestinal barrier by maintaining normal intestinal permeability in wild-type nematodes. Moreover, LAB prevented GO damage on the functions of both primary and secondary targeted organs in exposed nematodes with mutations of susceptible genes (*sod-2*, *sod-3*, *gas-1*, and *aak-2*) to GO toxicity by sustaining normal intestinal permeability. LAB also sustained the normal defecation behavior in both wild-type nematodes and nematodes with mutations of susceptible genes. Therefore, the beneficial role of LAB against GO toxicity under different genetic backgrounds may be due to the combinational effects on intestinal permeability and defecation behavior. Moreover, the beneficial effects of LAB against GO toxicity was dependent on the function of ACS-22, homologous to mammalian FATP4 to mammalian FATP4. Our study provides highlight on establishment of pharmacological strategy to protect intestinal barrier from toxicity of GO.

Graphene oxide (GO), a member of the graphene, is a single-atom thick sheet of sp^2^-bonded carbon atoms in a closely packed honeycomb two-dimensional lattice with unique physical, chemical, electrical, and mechanical properties^1^. Although GO is potentially used in biomedicine including drug delivery, imaging, and photocatalysts^2^,^3^,^4^, some studies have demonstrated the adverse effects of GO on human health and environmental organisms^4^,^5^. Besides the *in vitro* toxicity^6^,^7^,^8^, GO exposure also led to a series of *in vivo* toxicity such as pulmonary toxicity, reproductive toxicity in mammals^4^,^9^,^10^,^11^. It was also reported that GO exposure may result in the genotoxicity on organisms^12^,^13^. Moreover, it was reported that GO exposure could cause the increase in the villus length and width in duodenum regions, implying the remodeled intestinal villi^14^. So far, several cellular mechanisms have been raised to explain the GO toxicity: (1) direct contact interaction of ultra sharp edges of GO with cell membrane^15^,^16^, (2) induction of reactive oxygen species (ROS) production^17^,^18^, and (3) wrapping cells or microorganisms and aggregation in the culture medium^19^,^20^.

*Caenorhabditis elegans*, a free-living and abundant soil nematodes, is well-characterized structurally and genetically^21^,^22^. The conserved property of basic physiological processes, signaling pathways, genetic control, and stress responses between *C. elegans* and mammals or humans enables the *C. elegans* a useful non-mammalian alternative toxicity assay model^5^,^23^. In *C. elegans*, engineered nanomaterials (ENMs) can be translocated into the primary targeted organs (such as intestine) and/or the secondary targeted organs (such as neuron and reproductive organs)^24^,^25^,^26^,^27^. Moreover, our previous study has indicated the crucial role of biological barrier of intestine to be against the possible toxicity from ENMs in nematodes^28^. Structurally, the intestine contains the apical domain including brush border and terminal web, the basolateral domain, and the apical junctions joining one enterocyte to its partner and to adjacent ints^29^. In *C. elegans*, intestine is the organ responsible for food digestion, assimilation and metabolism of macromolecules, defecation, and stress response^29^. The motor program occurred in the intestine participates in the control of defecation behavior^29^. *C. elegans* can be successfully used for the toxicity assessment and toxicological study of carbon-based ENMs such as graphite, multi-walled carbon nanotubes (MWCNTs), and fullerenol^30^,^31^,^32^,^33^. Previous studies have further demonstrated that GO exposure could result in toxicity on the functions of both primary (such as intestine) and secondary (such as neuron and reproductive organs) targeted organs in nematodes^34^,^35^,^36^. In addition, the observed GO toxicity may be largely due to the combinational effects of oxidative stress, impaired intestinal barrier, and prolonged defecation cycle length in nematodes^37^.

In order to reduce the toxicity of GO, besides the chemical modifications^38^,^39^, recently it has been further indicated that specific pharmacological administration could be employed to be against the nanotoxicity^40^,^41^. Lactic acid bacteria (LAB) is the potential probiotic bacteria, and generally considered as safe and useful for food and feed fermentation^42^. In *C. elegans*, feeding with specific LAB strains could be resistant to pathogenic infection^43^,^44^. We assume that administration with LAB may be helpful for maintaining the functional state of intestinal barrier so as to be against the toxicity of GO in nematodes. Thus, in the present study, we employed the *in vivo C. elegans* assay system to investigate the possible beneficial effect of LAB against the GO toxicity and the underlying mechanisms. Our study will provide the insights on the establishment of pharmacological strategy in order to protect the intestinal barrier from the adverse effects of GO in organisms.

## Results

### Physicochemical properties of prepared GO

**Figure 1:**
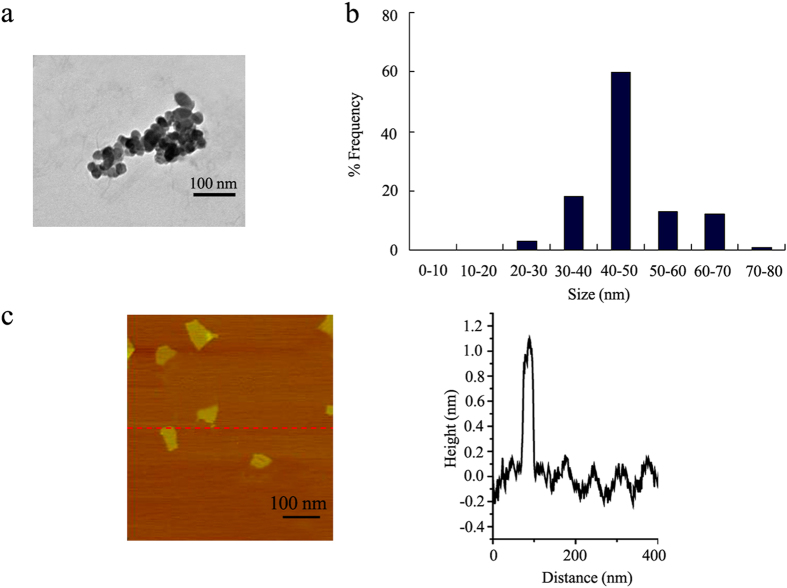
Physiochemical characterization of the GO. (**a**) TEM image of GO after sonification. (**b**) Size distribution of GO in K medium after sonification. (**c**) AFM analysis of GO.

The sizes of most of the GO in K-medium after sonication (40 kHz, 100 W, 30-min) were in the range of 40–50 nm (Fig. 1a,b). The GO aggregation size was 274 ± 72 nm (Fig. 1a). The atomic force microscopy (AFM) results for GO suggest its one-layer property (Fig. 1c). The height image from AFM assay indicates that the thickness of the prepared GO was about 1.0 nm in topographic height (Fig. 1c). Zeta potential of GO was −20.3 ± 1.6 mV. Raman spectroscopy measurement suggested the introduction of disorder into the graphite layer (Fig. S1). GO had a G band at 1597 cm^−1^ and a D band at 1352 cm^−1^ (Fig. S1).

X-ray photoelectron spectrum (XPS) analysis further indicate that GO had a C/O ratio of 2.32 (Fig. S2). The binding energy of C = C and C-C are assigned at 284.6 eV, 286.7 eV for C-O, and 288.8 eV for O = C (Fig. S2). The content of COOH in GO is 2.13%, and the content of OH group in GO is 50.35% (Fig. S2).

### Administration with LAB prevented the toxicity of GO on wild-type nematodes

**Figure 2:**
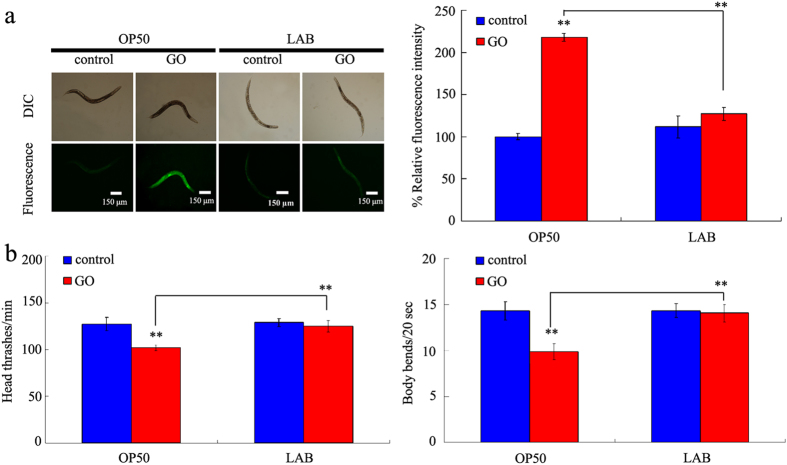
LAB administration prevented the toxicity of GO in wild-type nematodes. (**a**) LAB administration prevented the induction of intestinal ROS production induced by GO exposure in wild-type nematodes. (**b**) LAB administration prevented the toxicity of GO on locomotion behavior in wild-type nematodes. Locomotion behavior was assessed by the endpoints of head thrash and body bend. GO exposure concentration was 100 mg/L. The used LAB strain was *L. bulgaricus*. L4-larvae were pre-treated with LAB for 12 h, and then exposed to GO for 24 h at 20 °C. Bars represent means ± S.E.M. ^**^P < 0.01 *vs* control.

To determine the effect of LAB administration on toxicity of GO on the functions of primary targeted organs, we selected the endpoint of intestinal reactive oxygen species (ROS) production^45^. Previous study has suggested that acute exposure to 100 mg/L of GO caused the adverse effects on the functions of both primary and secondary targeted organs in nematodes^37^. Acute exposure to GO (100 mg/L) induced the significant intestinal ROS production compared with control in wild-type nematodes (Fig. 2a). In contrast, pretreatment with LAB (*L. bulgaricus*) significantly inhibited the induction of intestinal ROS production (Fig. 2a). LAB treatment alone did not induce the noticeable intestinal ROS production in wild-type nematodes (Fig. 2b).

To determine the effect of LAB administration on toxicity of GO on the functions of secondary targeted organs, we selected the endpoints or head thrash and body bend, which reflect the state of locomotion behavior of nematodes^32^. Acute exposure to GO (100 mg/L) significantly decreased the head thrash of body bend of nematodes compared with control in wild-type nematodes (Fig. 2b). Pretreatment with LAB significantly suppressed the decrease in head thrash or body bend observed in GO (100 mg/L) exposed wild-type nematodes (Fig. 2b). LAB treatment alone did not obviously affect the locomotion behavior of wild-type nematodes (Fig. 2b). These results suggest that pretreatment with LAB may be beneficial for being against the toxic effects of GO on the functions of both primary and secondary targeted organs in wild-type nematodes.

### Administration with LAB altered the translocation pattern of GO in wild-type nematodes

**Figure 3:**
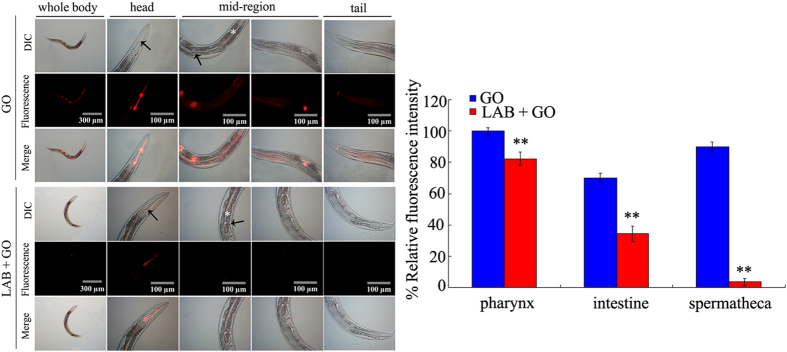
GO distribution in wild-type nematodes. GO-Rho B was used to visualize the distribution of GO in nematodes. The arrowheads indicate the pharynx and spermatheca, respectively, at the head region or mid-region of nematodes. The intestine (*) in the mid-region was also indicated. GO-Rho B exposure concentration was 100 mg/L. The used LAB strain was *L. bulgaricus*. L4-larvae were pre-treated with LAB for 12 h, and then exposed to GO-Rho B for 24 h at 20 °C. Bars represent means ± S.E.M. ^**^P < 0.01 *vs* GO.

Distribution or translocation is the key cellular basis for toxicity formation of ENMs including GO in nematodes^5^. After exposure, GO could be distributed in both the primary targeted organs such as intestine and pharynx and the secondary targeted organs such as the reproductive organ of spermatheca in wild-type nematodes (Fig. 3). However, this GO translocation pattern was obviously altered by pretreatment with LAB. After pretreatment with LAB, GO was mainly distributed in the pharynx and intestine, and no signals were detected in the secondary targeted organs of wild-type nematodes (Fig. 3). Compared with the distribution of GO-Rho B in wild-type nematodes, exposure to Rho B caused the relatively equable distribution of fluorescence in tissues of wild-type nematodes (Fig. S3).

### Administration with LAB was helpful for maintaining the normal state of intestinal permeability in GO exposed wild-type nematodes

**Figure 4:**
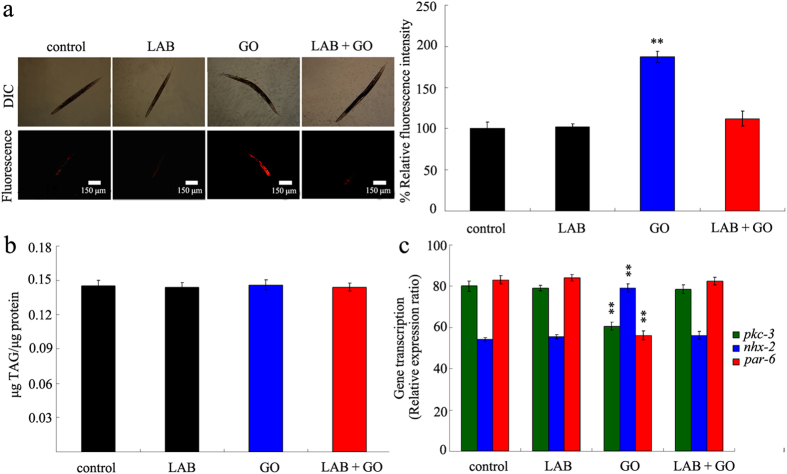
LAB administration maintained the normal intestinal permeability in GO exposed wild-type nematodes. (**a**) Nile red staining results. (**b**) Comparison of triglyceride content. (**c**) Comparison of gene expression patterns of *pkc-3*, *nhx-2*, and *par-6* genes. GO exposure concentration was 100 mg/L. The used LAB strain was *L. bulgaricus*. L4-larvae were pre-treated with LAB for 12 h, and then exposed to GO for 24 h at 20 °C. Bars represent means ± S.E.M. ^**^P < 0.01 *vs* control.

To determine the underlying cellular mechanism for altered translocation pattern of GO in LAB pretreated nematodes, we further investigated the permeability of primary targeted organs for GO exposed nematodes. We explored the lipophilic fluorescent dye, Nile Red to stain GO exposed nematodes. LAB pretreatment alone did not obviously affect the fluorescence intensity of Nile Red in intestine of wild-type nematodes (Fig. 4a). However, we found that exposure to GO (100 mg/L) induced the significantly enhanced fluorescence intensity of Nile Red in intestine compared with control in wild-type nematodes (Fig. 4a). In contrast, pretreatment with LAB noticeably blocked the increase in fluorescence intensity of Nile Red in intestine of wild-type nematodes (Fig. 4a). Considering the fact that Nile Red can also be used to label fat storage^46^, we further analyzed the triglyceride content of nematodes. LAB pretreatment or GO (100 mg/L) exposure did not significantly influence the triglyceride content compared with control in wild-type nematodes (Fig. 4b). After LAB pretreatment, the GO (100 mg/L) exposed wild-type nematodes also showed the similar triglyceride content to that in control wild-type nematodes (Fig. 4b). These results suggest that LAB pretreatment may potentially block the formation of hyper-permeable intestinal barrier in GO exposed nematodes.

Previous study has demonstrated that GO exposure could dysregulated the expression of some genes required for the control of intestinal development, such as *pkc-3*, *nhx-2*, and *par-6* genes^37^. In *C. elegans*, *pkc-3* gene encodes an atypical protein kinase, *nhx-2* gene encodes a sodium/proton exchanger, and *par-6* gene encodes a PDZ-domain-containing protein. *pkc-3*, *nhx-2*, and *par-6* genes are required for the control of development of intestinal microvilli in nematodes^29^. LAB pretreatment alone did not significantly affect the expression patterns of *pkc-3*, *nhx-2*, and *par-6* genes in wild-type nematodes (Fig. 4c). Exposure to GO (100 mg/L) significantly decreased the expression levels of *pkc-3* and *par-6* genes, and increased the expression level of *nhx-2* gene in wild-type nematodes (Fig. 4c). In contrast, pretreatment with LAB obviously inhibited the decrease in expression levels of *pkc-3* and *par-6* genes, and suppressed the increase in expression level of *nhx-2* gene in wild-type nematodes (Fig. 4c).

### Administration with LAB maintained the normal defecation behavior in GO exposed wild-type nematodes

**Figure 5:**
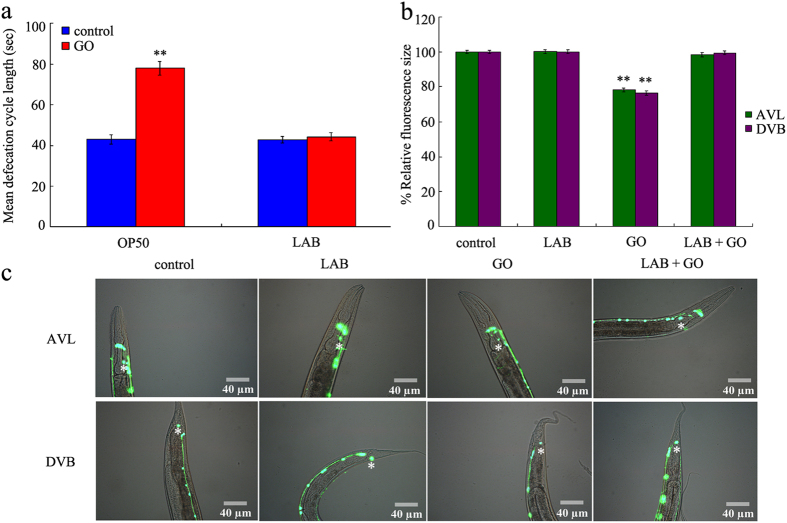
LAB administration maintained the normal defecation behavior in GO exposed wild-type nematodes. (**a**) Comparison of mean defecation cycle length. (**b**) Comparison of relative fluorescence size of cell body for AVL or DVB neurons. (**c**) Pictures showing the AVL and DVB neurons. Asterisks indicate the positions of AVL or DVB neurons. GO exposure concentration was 100 mg/L. The used LAB strain was *L. bulgaricus*. L4-larvae were pre-treated with LAB for 12 h, and then exposed to GO for 24 h at 20 °C. Bars represent means ± S.E.M. ^**^P < 0.01 *vs* control.

In *C. elegans*, besides the induction of hyper-permeable intestinal barrier, formation of abnormal defecation behavior is another important cellular basis for the toxicity of ENMs^5^. Exposure to GO (100 mg/L) significantly increased the mean defecation cycle length of wild-type nematodes (Fig. 5a). In contrast, LAB pretreatment noticeably recovered the toxic effect of GO (100 mg/L) on defecation behavior in wild-type nematodes (Fig. 5a). LAB pretreatment alone did not obviously influence the defecation behavior (Fig. 5a).

In *C. elegans*, AVL and DVB neurons are involved in the control of defecation behavior^25^. Exposure to GO (100 mg/L) significantly reduced the relative fluorescence size of cell body for AVL or DVB neurons (Fig. 5b,c). In contrast, LAB pretreatment noticeably suppressed the reduction in relative fluorescence size of cell body for AVL or DVB neurons induced by GO (100 mg/L) exposure (Fig. 5b,c). LAB pretreatment alone did not obviously affect the development of AVL or DVB neurons in wild-type nematodes (Fig. 5b,c).

### Administration with LAB prevented the toxicity of GO on nematodes with mutations of susceptible gene

**Figure 6:**
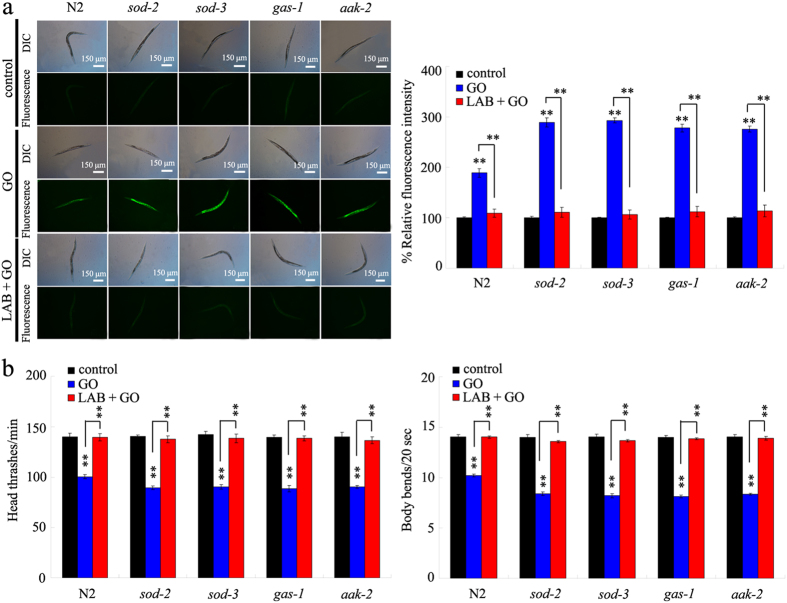
LAB administration prevented the toxicity of GO in nematodes with mutations of susceptible gene. (**a**) LAB administration prevented the induction of intestinal ROS production induced by GO exposure in nematodes with mutations of susceptible gene. (**b**) LAB administration prevented the toxicity of GO on locomotion behavior in nematodes with mutations of susceptible gene. Locomotion behavior was assessed by the endpoints of head thrash and body bend. GO exposure concentration was 100 mg/L. The used LAB strain was *L. bulgaricus*. L4-larvae were pre-treated with LAB for 12 h, and then exposed to GO for 24 h at 20 °C. Bars represent means ± S.E.M. ^**^P < 0.01 *vs* control (if not specially indicated).

Previous study has suggested that mutations of some genes required for the control of oxidative stress, such as *sod-2*, *sod-3*, *gas-1*, or *aak-2* gene, caused the susceptible property of nematodes to GO toxicity^35^. In *C. elegans*, *sod-2* and *sod-3* genes encode the mitochondrial manganese-superoxide dismutases, *gas-1* gene encodes a subunit of mitochondrial complex I, and *aak-2* gene encodes a catalytic alpha subunit of AMP-activated protein kinase. Mutation of *sod-2*, *sod-3*, *gas-1*, or *aak-2* gene led to the more severe induction of intestinal ROS production, and decrease in locomotion behavior in GO (100 mg/L) exposed nematodes compared with GO (100 mg/L) exposed wild-type N2 (Fig. 6). In contrast, we found that LAB pretreatment could still effectively suppress the induction of intestinal ROS production, and the decrease in locomotion behavior in GO (100 mg/L) exposed *sod-2*, *sod-3*, *gas-1*, or *aak-2* mutant nematodes (Fig. 6). These results imply that, under the *sod-2*, *sod-3*, *gas-1*, or *aak-2* mutation background, LAB pretreatment may have the beneficial effect in being against the GO toxicity in nematodes.

### Administration with LAB prevented the damage of GO exposure on intestinal barrier in nematodes with mutations of susceptible gene

**Figure 7:**
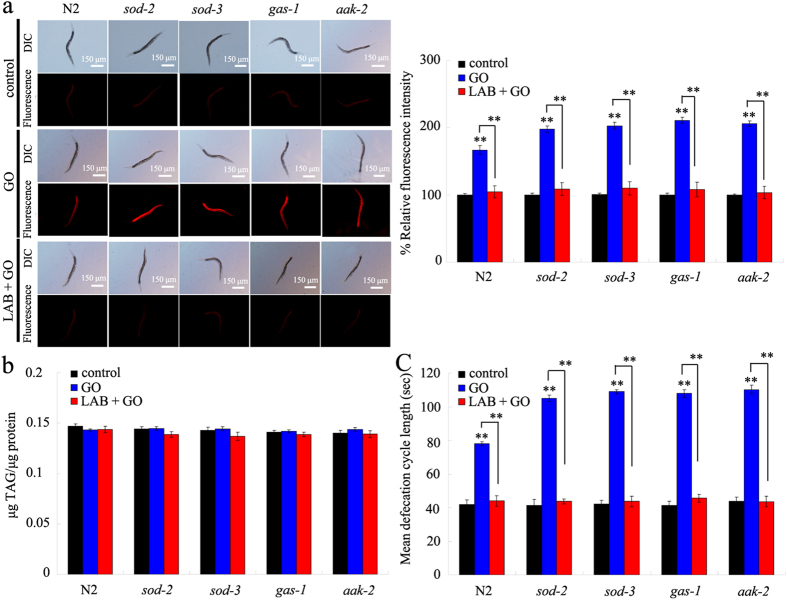
LAB administration prevented the damage of GO exposure on intestinal permeability and defecation behavior in nematodes with mutations of susceptible gene. (**a**) Nile red staining results. (**b**) Comparison of triglyceride content. (**c**) Comparison of mean defecation cycle length. GO exposure concentration was 100 mg/L. The used LAB strain was *L. bulgaricus*. L4-larvae were pre-treated with LAB for 12 h, and then exposed to GO for 24 h at 20 °C. Bars represent means ± S.E.M. ^**^P < 0.01 *vs* control (if not specially indicated).

To determine the cellular basis for the potential of LAB pretreatment in preventing GO toxicity in nematodes with mutations of susceptible gene, we investigated the intestinal permeability in GO exposed nematodes with mutations of susceptible gene. The *sod-2*, *sod-3*, *gas-1*, and *aak-2* mutants had the similar Nile Red staining results and triglyceride content to those in wild-type N2 nematodes (Fig. 7a,b), suggesting that mutations of *sod-2*, *sod-3*, *gas-1*, or *aak-2* gene did not obviously affect the intestinal permeability of nematodes. Although the GO (100 mg/L) exposed *sod-2*, *sod-3*, *gas-1*, or *aak-2* mutants had the similar triglyceride content to that in wild-type N2 nematodes without GO exposure, GO (100 mg/L) exposed *sod-2*, *sod-3*, *gas-1*, or *aak-2* mutants had the more increased relative fluorescence intensity of Nile Red signals in intestine than GO (100 mg/L) exposed wild-type N2 nematodes (Fig. 7a,b). In contrast, we further found that LAB pretreatment could significantly inhibit the increase in relative fluorescence intensity of Nile Red signals in intestine of GO (100 mg/L) exposed *sod-2*, *sod-3*, *gas-1*, or *aak-2* mutants (Fig. 7a,b).

### Administration with LAB prevented the damage of GO exposure on defecation behavior in nematodes with mutations of susceptible gene

To further determine the cellular basis for the potential of LAB pretreatment in preventing GO toxicity in nematodes with mutations of susceptible gene, we also investigated the defecation behavior in GO exposed nematodes with mutations of susceptible gene. The *sod-2*, *sod-3*, *gas-1*, and *aak-2* mutants had the similar mean defecation cycle length to those in wild-type N2 nematodes (Fig. 7c), suggesting that mutations of *sod-2*, *sod-3*, *gas-1*, or *aak-2* gene did not noticeably influence the defecation cycle of nematodes. The GO (100 mg/L) exposed *sod-2*, *sod-3*, *gas-1*, or *aak-2* mutants had the more prolonged mean defecation cycle length than GO (100 mg/L) exposed wild-type N2 nematodes (Fig. 7c). In contrast, LAB pretreatment could significantly suppress the increase in mean defecation cycle length in GO (100 mg/L) exposed *sod-2*, *sod-3*, *gas-1*, or *aak-2* mutants (Fig. 7c).

### Effects of *acs-22* mutation on beneficial effects of LAB administration against GO toxicity

**Figure 8:**
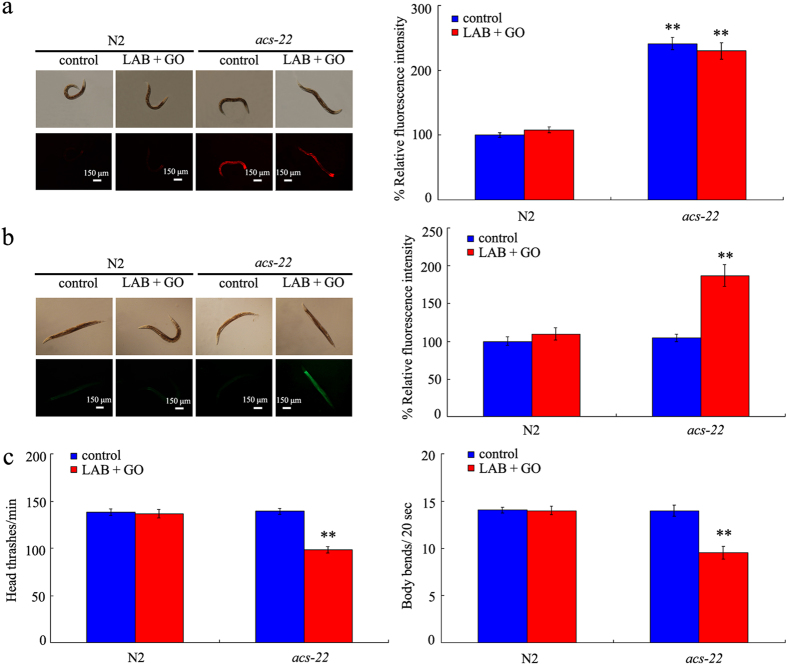
Effects of *acs-22* mutation on beneficial effects of LAB administration against GO toxicity. (**a**) Effects of *acs-22* mutation on beneficial effects of LAB administration against GO-induced enhancement of intestinal permeability as indicated by Nile Red staining results. (**b**) Effects of *acs-22* mutation on beneficial effects of LAB administration against GO-induced intestinal ROS production. (**c**) Effects of *acs-22* mutation on beneficial effects of LAB administration against GO-induced decrease in locomotion behavior. GO exposure concentration was 100 mg/L. The used LAB strain was *L. bulgaricus*. L4-larvae were pre-treated with LAB for 12 h, and then exposed to GO for 24 h at 20 °C. Bars represent means ± S.E.M. ^**^P < 0.01 *vs* N2.

In *C. elegans*, *acs-22* gene encodes a protein homologous to mammalian FATP4 (fatty acid transport protein 4), a key factor involved in forming the stratum corneum barrier^47^. After exposure, we found that GO significantly decreased the expression level of *acs-22* gene compared with control (Fig. S4). In contrast, LAB pretreatment could maintain the normal expression of acs-22 gene in nematodes exposed to GO (Fig. S4). In nematodes, mutation of *acs-22* gene induced the significant increase in relative fluorescence intensity of Nile Red signals in intestine of animals (Fig. 8a). In contrast, mutation of *acs-22* gene did not obviously alter the triglyceride content (data not shown), induce the significant intestinal ROS production (Fig. 8b), and influence the locomotion behavior of animals (Fig. 8c). These results imply the possible involvement of *acs-22* gene in the control of intestinal permeability in nematodes.

Moreover, after LAB administration, we still could observe the significant increase in relative fluorescence intensity of Nile Red signals in intestine, induction of intestinal ROS production, and decrease in locomotion behavior in GO exposed *acs-22* mutant nematodes (Fig. 8). Meanwhile, LAB administration did not alter the triglyceride content in *acs-22* mutant exposed to GO (data not shown). Therefore, the beneficial effects of LAB administration against GO toxicity may be dependent on the function of ACS-22. In nematodes, LAB administration may exert its beneficial effects on maintaining the normal intestinal permeability through influencing the function of ACS-22.

## Discussion

In this study, we investigated the possible beneficial effects of LAB treatment in reducing *in vivo* GO toxicity. Considering the important contribution of *C. elegans* to nanotoxicology study^5^, we employed the non-mammalian alternative toxicity assay model of *C. elegans* as the *in vivo* assessment system. Considering the multiple beneficial effects of *L. bulgaricus* on organisms such as suppressing inflammation and gastrointestinal well-being^48^,^49^, we selected the *L. bulgaricus* as the testing LAB bacteria. In the present study, we mainly selected the intestinal ROS production and locomotion behavior as the toxicity assessment endpoints to reflect the functions of primary and secondary targeted organs, respectively. To evaluate the function of secondary targeted organs, we did not select the endpoint of brood size, because feeding with LAB might cause the laid eggs to be hatched and arrested as L1-larvae^50^. In this study, we investigated the effects of LAB pretreatment on GO toxicity in order to determine the possible prevention function of LAB on GO toxicity in nematodes.

In *C. elegans*, it was reported that LAB protected animals from enterotoxigenic *Escherichia coli*-caused death by inhibiting enterotoxin gene expression of the pathogen^51^. One of the important cellular mechanisms for GO toxicity is that GO exposure may potentially induce the significant ROS production^17^,^18^. In the present study, we further show that LAB pretreatment could effectively prevent the toxicity of GO exposure in inducing intestinal ROS production and decreasing locomotion behavior in nematodes (Fig. 2). Our data confirmed the function of LAB treatment in preventing nematodes from the damage of oxidative stress^52^. Moreover, our results suggest that LAB pretreatment could potentially maintain the normal functions of both primary and secondary targeted organs in GO exposed nematodes.

The altered translocation pattern of GO in LAB pretreated nematodes implies the key cellular mechanism for the beneficial effects of LAB pretreatment against GO toxicity. Nematodes without LAB pretreatment exhibited the distribution of GO in both primary and secondary targeted organs; however, nematodes with LAB pretreatment showed the distribution of GO mainly in pharynx and intestine (Fig. 3). The crucial cellular mechanisms for GO toxicity is the direct contact interaction of GO with cell membrane^15^,^16^. Our results suggest that LAB pretreatment may block the translocation of GO into the secondary targeted organs through the intestinal barrier in nematodes. The further experimental evidence supported this assumption. LAB pretreatment maintained the normal intestinal permeability in GO exposed wild-type nematodes (Fig. 4a,b). Especially, LAB pretreatment prevented the dysregulation of genes involved in the control of intestinal development induced by GO exposure in nematodes (Fig. 4c). These results suggest that LAB pretreatment may block the bioavailability of GO to cells, as well as the possible charge transfer from GO to cells, in the body of nematodes by sustaining the normal intestinal permeability. These data support the potential of LAB treatment in gastrointestinal well-being maintenance reported previously^49^. That is, LAB pretreatment may be helpful for preventing the damage of GO exposure on intestinal development as observed previously in nematodes and mice^14^,^37^. In contrast, the inhibition of ROS production induced by GO exposure may the indirect result of LAB pretreatment. In addition, considering the fact that LAB pretreatment could effectively suppress the translocation of GO into the reproductive organs in nematodes (Fig. 3), our results imply that LAB pretreatment may suppress the bioavailability of GO to germ cells in the reproductive organs and inhibit the genotoxicity potentially induced by GO in nematodes.

Previous study has identified some susceptible genes such as *sod-2*, *sod-3*, *gas-1*, and *aak-2* genes to GO toxicity in nematodes^35^. Some of these genes encode the molecular regulation machinery for oxidative stress. For example, *gas-1* gene encodes a subunit of mitochondrial complex I in nematodes. Previous studies have suggested that treatment with antioxidant such as vitamin E or paeonol with the function against oxidative damage could inhibit the toxicity of ENMs such as Al_2_O_3_-NPs or MWCNTs^40^,^41^. However, under such a genetic background, the normally used antioxidants such as vitamin E can not exert the anticipated beneficial effects in being against GO toxicity (data not shown). Especially, treatment with high doses of some antioxidants such as vitamine E may have the adverse effects on organisms^40^,^53^. Interestingly, we found that LAB pretreatment could also effectively prevent the damage of GO exposure on both the primary and the secondary targeted organs in *sod-2*, *sod-3*, *gas-1*, or *aak-2* mutants (Fig. 6).

The key cellular mechanism for the observed beneficial effects of LAB pretreatment against GO toxicity may be also due to the maintenance of intestinal permeability in GO exposed *sod-2*, *sod-3*, *gas-1*, or *aak-2* mutants. We observed that the normal intestinal permeability in GO exposed *sod-2*, *sod-3*, *gas-1*, or *aak-2* mutants could be well sustained by LAB pretreatment (Fig. 7a,b). Previous study has also suggested that administration with LAB could increase the lifespan of nematodes^52^. These results further imply that the establishment a blockage between GO and targeted organs may be a very effective strategy against the GO toxicity during their long-term exposure. Previous study has suggested that PEGylation could only partially improve the biocompatibility of carbon-based ENMs in mice after long-term (2-month) exposure^54^. With the respect to the molecular mechanism for the observed beneficial effects of LAB pretreatment against GO toxicity, we hypothesize that LAB pretreatment may exert its beneficial effects in maintaining the intestinal permeability and in inhibiting GO toxicity through influencing the function of ACS-22 in nematodes (Fig. 8). Therefore, the combinational use of effective pharmacological administration and chemical surface modification should be carefully considered in order to prevent the possible toxicity of specific ENMs on organisms.

Besides the maintenance of intestinal permeability, LAB pretreatment was also observed to have the function in sustaining the normal defecation behavior in wild-type nematodes (Fig. 5a). The possible cellular mechanism about this may be also due to the maintenance of intestinal permeability in nematodes, and such a mechanism could further protect the AVL and DVB neurons from the damage of GO exposure (Fig. 5b,c). AVL neurons in the head and DVB neurons in the tail are required for the control of defecation behavior in nematodes^25^. Under the susceptible genetic background, we further observed the maintenance of normal defecation behavior in nematodes (Fig. 7c). Therefore, the beneficial role of LAB pretreatment against GO toxicity may be at least due to the combinational effects on intestinal permeability and defecation behavior in nematodes.

In conclusion, LAB pretreatment could effectively suppress the toxicity of GO exposure on the function of both primary and secondary targeted organs in nematodes. One of the main cellular mechanisms for the beneficial effects of LAB pretreatment is the maintenance of normal intestinal permeability in GO exposed nematodes. Another cellular mechanism for the beneficial effects of LAB pretreatment is the maintenance of normal defecation behavior in GO exposed nematodes. The combinational effects on intestinal permeability and defecation behavior by LAB pretreatment prevented the translocation of GO into the secondary targeted organs or bioavailability of GO to cells in the body through the intestinal barrier in nematodes. One of the important molecular mechanisms for the beneficial effects of LAB pretreatment is that LAB may exert its beneficial effects against GO toxicity through influencing the function of ACS-22 in nematodes. More interestingly, we found that the beneficial effects of LAB pretreatment against GO toxicity could also be observed in nematodes with mutations of susceptible genes.

## Methods

### Reagents and preparations of GO

GO was prepared from natural graphite powder using modified Hummer’s method^55^,^56^. Graphite (2 g) and sodium nitrate (1 g) were added into a 250-mL flask. After addition of concentrated H_2_SO_4_ (50 mL) on ice, KMnO4 (7 g) was added to the mixture. Again, 90 mL of H_2_O was slowly dripped into the paste to cause an increase in temperature to 70 °C after temperature of the mixture warmed to 35 °C. After stirring the diluted suspension at 70 °C for another 15 min, the suspension was treated with a mixture of 7 mL of 30% H_2_O_2_ and 55 mL of H_2_O. The resulting warm suspension was filtered to obtain a yellow-brown filter cake. The filter cake was washed for three times with a solution of 3% HCl, followed by drying at 40 °C for 24 h. GO was finally obtained by ultrasonication of as-made graphite oxide in water for 1 h.

GO was sonicated for 30-min (40 kHz, 100 W), and dispersed in K medium to prepare the stock solution (1 mg/mL). The stock solution was diluted to the used concentration (100 mg/L) with K medium just prior to exposure^37^. All the other chemicals were obtained from Sigma-Aldrich (St. Louis, MO, USA).

### Characterization of GO

GO was characterized by transmission electron microscopy (TEM, JEM-200CX, JEOL, Japan), AFM (SPM-9600, Shimadzu, Japan), and Raman spectroscopy (Renishaw Invia Plus laser Raman spectrometer, Renishaw, UK). Zeta potential analyzed by the Nano Zetasizer using a dynamic light scattering (DLS) technique. To perform AFM measurement, a few drops of the GO suspension was pipetted on Si substrates, and then the substrates were air-dried and placed under the AFM tip for morphology analysis. Elemental composition analysis was carried out by XPS (AXIS Ultra instrument, Kratos, UK).

### *C. elegans* strain preparation

Nematodes used in the present study were wild-type N2, and mutants of *sod-2(ok1030)*, *sod-3(gk235)*, *gas-1(fc21)*, *aak-2(ok524)*, and *acs-22(tm3236)*. Some strains were originally obtained from the *Caenorhabditis* Genetics Center (funded by NIH Office of Research Infrastructure Programs (P40 OD010440)). Nematodes were maintained on nematode growth medium (NGM) plates seeded with *Escherichia coli* OP50 at 20 °C as described^21^. Gravid nematodes were washed off the plates into centrifuge tubes, and lysed with a bleaching mixture (0.45 M NaOH, 2% HOCl). Age synchronous populations of L4-larvae were obtained as described^57^. Exposure to GO was performed from day-1 adult for 24 h in 12-well sterile tissue culture plates at 20 °C in the presence of food (OP50). The exposed nematodes were used for toxicity assessment with the aid of intestinal ROS production and locomotion behavior as the endpoints.

### LAB administration

*Lactobacillus bulgaricus* bacteria were grown in de Man, Rogosa, and Sharpe (MRS) medium (Difco, Detroit, MI) at 37 °C for 24 h, and the bacteria were seeded on modified NGM plates (peptone free NGM). To examine the prevention effects of LAB on the toxicity of GO, L4-larvae were pre-treated on modified NGM plates fed with lawns of *L. bulgaricus* for 12 h at 20 °C. And then, the nematodes were exposed to GO by transferring the examined nematodes from the modified NGM plates fed with *L. bulgaricus* to wells of sterile tissue culture plates containing GO at 20 °C. After 24 h, the nematodes were used for the toxicity assessment.

### Toxicity assessment

The method for ROS production was performed as described^58^,^59^. The examined nematodes were transferred to 1 μM of 5′,6′-chloromethyl-2′,7′dichlorodihydro-fluorescein diacetate (CM-H_2_DCFDA; Molecular Probes) in 12-well sterile tissue culture plates to pre-incubate for 3 h at 20 °C in the dark, and then mounted on 2% agar pads for examination at 488 nm of excitation wavelength and 510 nm of emission filter with a laser scanning confocal microscope (Leica, TCS SP2, Bensheim, Germany). Relative fluorescence intensity of intestine was semi-quantified, and the semiquantified ROS was expressed as relative fluorescence units (RFU). Thirty nematodes were examined per treatment, and three replicates were performed.

Locomotion behavior of nematodes was assessed by endpoints of head thrash and body bend as described^60^,^61^. A head thrash was defined as a change in the direction of bending at the mid body, and a body bend was counted as a change in the direction of the part of the nematodes corresponding to the posterior bulb of the pharynx along the *y* axis, assuming that nematode was traveling along the *x* axis. Thirty nematodes were examined per treatment, and three replicates were performed.

### Distribution of GO in nematodes

To investigate the distribution of GO in nematodes, Rho B was loaded on GO by mixing Rho B solution (1 mg/mL, 0.3 mL) with an aqueous suspension of GO (0.1 mg/mL, 5 mL) basically as previously described^35^. Unbound Rho B was removed by dialysis against distilled water over 72 h. The resulting GO-Rho B was stored at 4 °C. The examined nematodes were incubated with GO-Rho B for 3 h, and washed with M9 buffer. Nematodes were then observed under a laser scanning confocal microscope (Leica, TCS SP2, Bensheim, Germany). Relative fluorescence intensity of GO-Rho B in pharynx and intestine (the primary targeted organs) and spermatheca (the secondary targeted organs) was examined. Ten nematodes were examined per treatment, and three replicates were performed. Rho B was used as a control.

### Nile Red staining

The methods were performed as described previously^35^. Nile Red (Molecular Probes, Eugene, OR) was dissolved in acetone to produce a 0.5 mg/mL stock solution and stored at 4 °C. Stock solution was freshly diluted in 1× PBS to 1 μg/mL, and 150 μL of the diluted solution was used for Nile Red staining. Thirty nematodes were examined per treatment, and three replicates were performed.

### Analysis of triglyceride content

Lipid of nematodes was extracted by the method as described previously^62^. The triglyceride content was measured using an enzymatic kit (Wako Triglyceride E-test, Wako Pure Chemical Ltd., Osaka, Japan). Ten replicates were performed.

### Defecation behavior analysis and fluorescent images of neurons controlling the defecation behavior

The method was performed as described previously^63^. To assay mean defecation cycle length, individual animal was examined for a fixed number of cycles, and a cycle period was defined as the interval between initiations of two successive posterior body-wall muscle contraction steps. Thirty nematodes were used for each mean defecation cycle length assay, and three replicates were performed.

The fluorescent images of AVL and DVB neurons controlling defecation behavior were captured with a Zeiss Axiocam MRm camera on a Zeiss Axioplan 2 Imaging System using SlideBook software (Intelligent Imaging Innovations). Images were acquired with a Quantix cooled charge-coupled device (CCD) camera, and illumination was provided by a 175 W xenon arc lamp and GFP filter sets. The relative sizes of fluorescent puncta for cell bodies of AVL and DVB neurons were measured as the maximum radius for assayed fluorescent puncta. The relative sizes of fluorescent puncta for cell bodies of AVL and DVB neurons were examined in at least 20 nematodes, and three replicates were performed.

### Reverse-transcription and quantitative real-time polymerase chain reaction (PCR)

Total RNA was extracted using RNeasy Mini Kit (Qiagen). Purity and concentration of RNA were evaluated by OD260/280 in a spectrophotometer. Total RNAs were reverse transcribed using the PrimeScript ^TM^ RT reagent kit (Takara, Otsu, Shiga, Japan). After cDNA synthesis, real-time PCR was performed using SYBR Premix Ex Taq™ (Takara) for amplification of the PCR products. The *tba-1* gene was chosen as a reference gene. All reactions were performed in triplicate with the same cDNA samples. The relative quantification of targeted genes in comparison to the reference *tba-1* gene encoding a tubulin protein was determined, and the final results were expressed as the relative expression ratio between targeted gene and reference gene. The designed primers for targeted genes and reference *tba-1* gene were shown in Table S1.

### Statistical analysis

All data in this article were expressed as means ± standard error of the mean (S.E.M.). Graphs were generated using Microsoft Excel (Microsoft Corp., Redmond, WA). Statistical analysis was performed using SPSS 12.0 (SPSS Inc., Chicago, USA). Differences between groups were determined using analysis of variance (ANOVA). Probability levels of 0.05 and 0.01 were considered statistically significant.

## Additional Information

**How to cite this article**: Zhao, Y. *et al.* Lactic Acid Bacteria Protects *Caenorhabditis elegans* from Toxicity of Graphene Oxide by Maintaining Normal Intestinal Permeability under different Genetic Backgrounds. *Sci. Rep.* **5**, 17233; doi: 10.1038/srep17233 (2015).

## Supplementary Material

Supplementary Information
