# Supplementary material for: Lactic Acid Bacteria Protects Caenorhabditis elegans from Toxicity of Graphene Oxide by Maintaining Normal Intestinal Permeability under different Genetic Backgrounds
Source: Sci Rep. 2015 Nov 27;5:17233. doi: 10.1038/srep17233 (PMC4661518; doi:10.1038/srep17233)
Supplement: Supplementary Information [file srep17233-s1.doc]

**Supporting Information:**

**
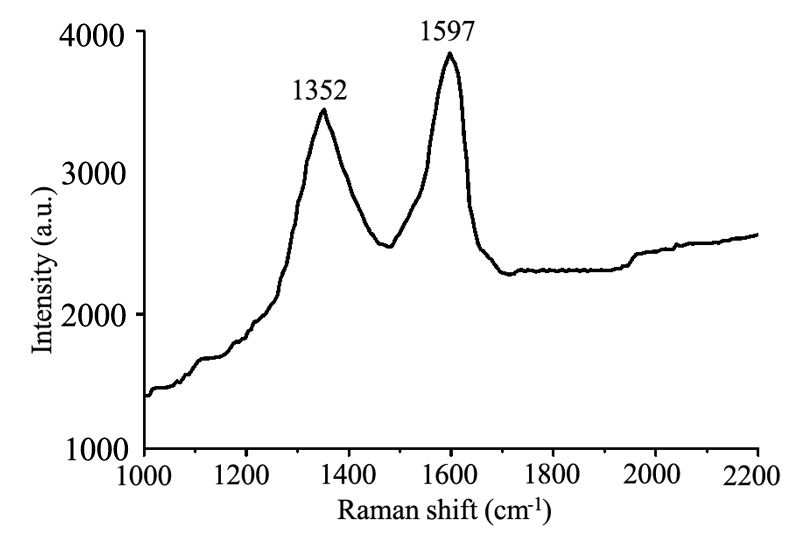
**

**Figure S1 Raman spectrum of GO.**

**
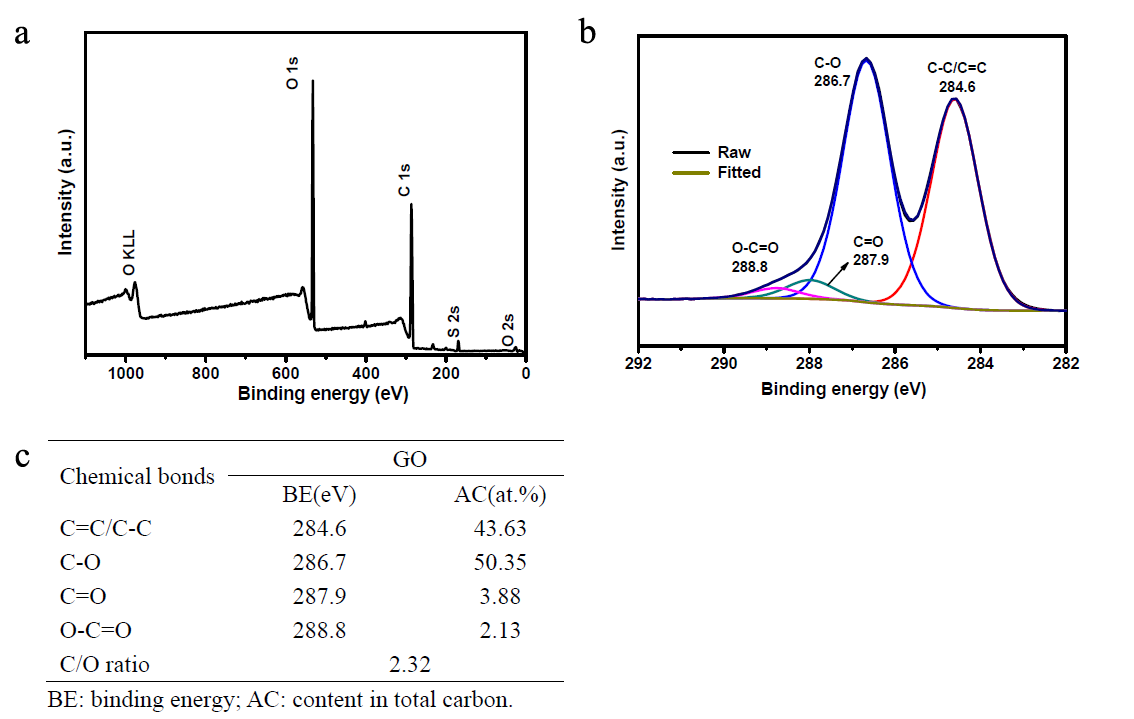
**

**Figure S2** **XPS data of GO.**  (a) XPS spectra of GO. (b) C 1s XPS spectra of GO. (c) Data analysis of the C1s XPS of GO.


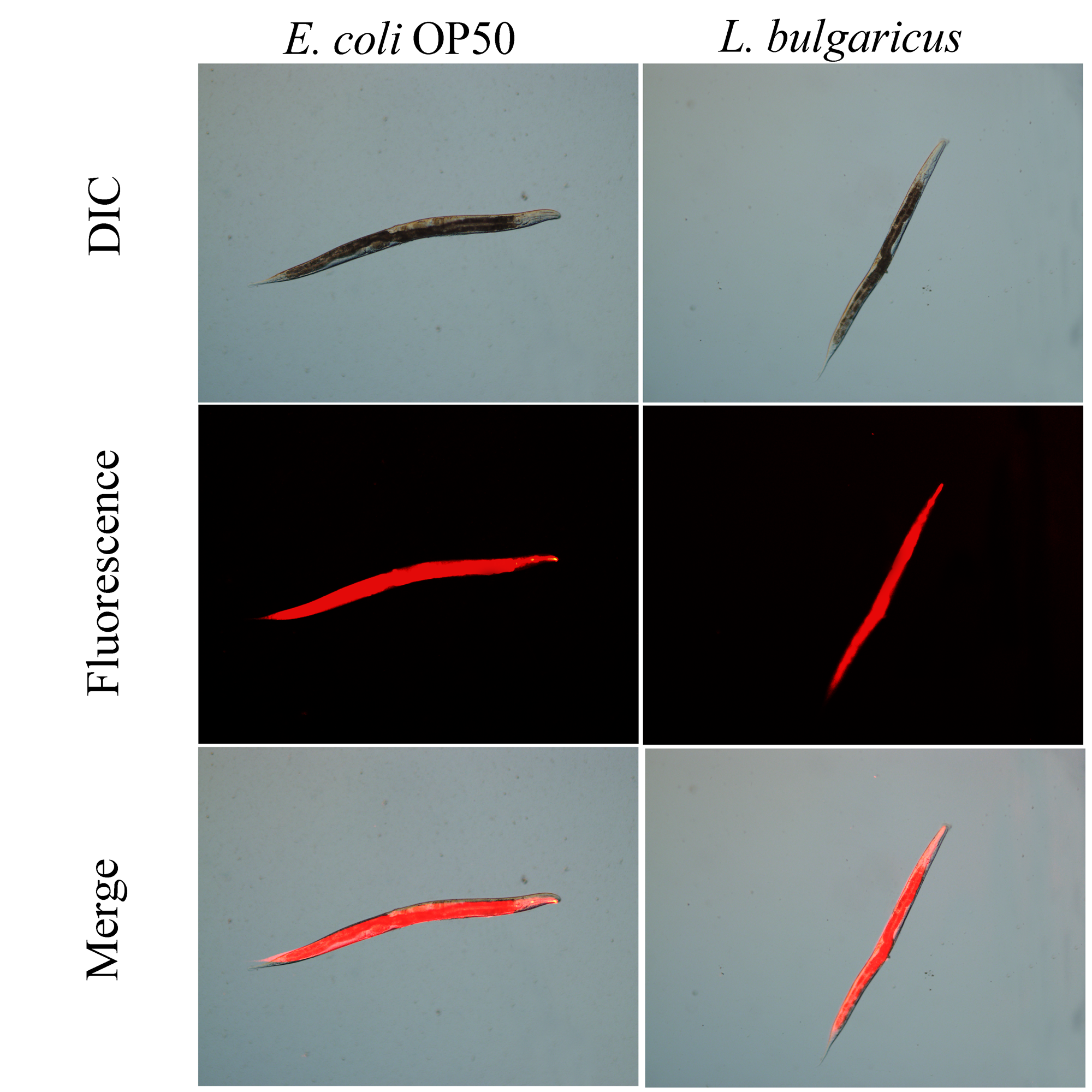


**Figure S3 Distribution of Rho B in wild-type nematodes.**


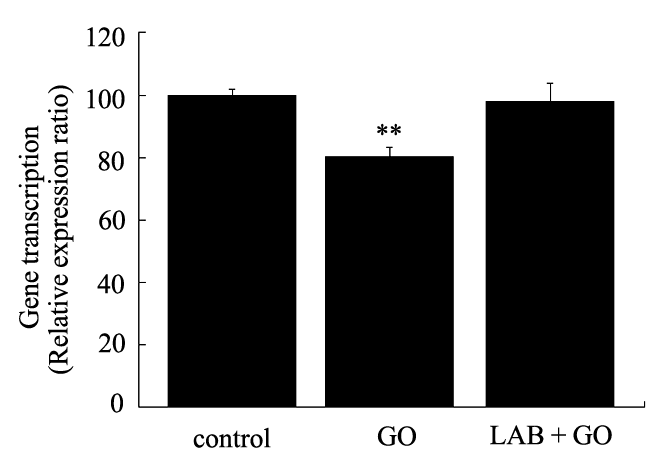


**Figure S4 Effects of GO exposure on *acs-22* gene expression.** GO exposure concentration was 100 mg/L. Young adults were exposed to GO for 24 h at 20°C. The used LAB strain was *L. bulgaricus*. L4-larvae were pre-treated with LAB for 12 h, and then exposed to GO for 24 h at 20°C. Bars represent means ± S.E.M. ^**^*P* < 0.01 *vs* control.

**Table S1 Primers used for quantitative real-time polymerase chain reaction (PCR)**

| Gene | Forward primer | Reverse primer |
| --- | --- | --- |
| *tba-1* | TCAACACTGCCATCGCCGCC | TCCAAGCGAGACCAGGCTTCAG |
| *pkc-3* | CGTCTCCGACATCATTAG | CAACTCGGCTTCTTGACT |
| *nhx-2* | GGAGCAGAATGTGAAGAA | GTGGCGGAAGTAGATAAA |
| *par-6* | ATTCTGCGTCTGGTGTCT | TTCCCTTCCATCGTTTAT |
| *acs-22* | CAATTTGGCTGGGACTTT | ACGAGCGGCTTTGAACAT |
